# Jian-Pi-Yi-Shen Formula Improves Adenine-Induced Chronic Kidney Disease *via* Regulating Tryptophan Metabolism and Aryl Hydrocarbon Receptor Signaling

**DOI:** 10.3389/fphar.2022.922707

**Published:** 2022-07-05

**Authors:** Xinhui Liu, Ruyu Deng, Yulian Chen, Shiying Huang, Jiandong Lu, Lin Zheng, Guoliang Xiong, Shunmin Li

**Affiliations:** ^1^ Department of Nephrology, Shenzhen Traditional Chinese Medicine Hospital, Guangzhou University of Chinese Medicine, Shenzhen, China; ^2^ Shenzhen Traditional Chinese Medicine Hospital Affiliated to Nanjing University of Chinese Medicine, Shenzhen, China; ^3^ The Fourth Clinical Medical College, Guangzhou University of Chinese Medicine, Shenzhen, China; ^4^ Shenzhen Key Laboratory of Hospital Chinese Medicine Preparation, Shenzhen Traditional Chinese Medicine Hospital, Guangzhou University of Chinese Medicine, Shenzhen, China

**Keywords:** chronic kidney disease, traditional Chinese medicine, Jian-Pi-Yi-Shen formula, tryptophan metabolism, aryl hydrocarbon receptor

## Abstract

Traditional Chinese medicine (TCM) is an important complementary and alternative branch of chronic kidney disease (CKD) therapy. Jian-Pi-Yi-Shen formula (JPYSF) is a TCM formula used for treating CKD with good efficacy. However, the underlying mechanisms of JPYSF in treating CKD remain to be elucidated. The purpose of the present study was to investigate the renoprotective effect and potential mechanism of JPYSF in treating CKD. CKD rat model was induced by feeding a diet containing 0.75% w/w adenine for 4 weeks. JPYSF was given by gavage every day, starting from the 3rd week of the adenine-containing diet and continuing for 4 weeks at the dose of 10.89 g/kg. Renal injury was evaluated by serum creatinine (Scr), blood urea nitrogen (BUN), histopathology, and fibrotic markers expression. Serum levels of tryptophan metabolites were detected by ultra-high performance liquid chromatography-tandem mass spectrometry (UHPLC-MS/MS). Aryl hydrocarbon receptor (AHR) signaling was tested by Western blot analysis. The results found that JPYSF treatment significantly lowered Scr and BUN levels, improved renal pathological injury, and down-regulated fibrotic markers expression in CKD rats. Furthermore, JPYSF significantly reduced the levels of 10 tryptophan metabolites in the serum of CKD rats and restored the level of tryptophan. Additionally, the kidney expression of AHR signaling was enhanced in CKD rats and was further suppressed in JPYSF treated rats. These results suggested that JPYSF protected against adenine-induced CKD via modulating tryptophan metabolism and AHR activation.

## Introduction

Chronic kidney disease (CKD) is a progressive disease characterized by structural and functional changes to the kidney due to various causes (Kalantar-Zadeh et al., 2021). The global burden of CKD is growing rapidly. In 2017, 697.5 million cases of all-stage CKD were recorded, for a global prevalence of 9.1%, which resulted in 1.2 million deaths (Bikbov et al., 2020). Traditional Chinese medicine (TCM) is an important complementary and alternative branch of CKD therapy and is being used by a great amount of the world’s population (Li and Wang, 2005). Jian-Pi-Yi-Shen Formula (JPYSF) contains 8 Chinese medicinal herbs and has been used in clinical treatment of CKD for decades with good efficacy. Our previous studies found that JPYSF could delay CKD progression in both 5/6 nephrectomy and adenine-induced CKD rat models (Liu et al., 2018; Lu et al., 2018; Liu et al., 2020; Liu et al., 2021a; Liu et al., 2021b). However, the mechanism of its efficacy remains to be further elucidated.

CKD is characterized by the accumulation of uremic toxins, which contribute to the pathogenesis of some of the systemic complications in CKD patients (Walker et al., 2020). Among uremic toxins, those derived from tryptophan metabolism are particularly of importance because they are associated with cardiovascular toxicity and are the ligands of the aryl hydrocarbon receptor (AHR) (Brito et al., 2017). Emerging evidence implicated that the AHR signaling pathway was activated both in CKD patients and in animal models of CKD (Dou et al., 2018; Ren et al., 2021). The AHR is a ligand-activated nuclear transcription factor that belongs to the basic helix-loop-helix protein superfamily. Physiologically, AHR is located in the cytoplasm and dimerizes with heat shock protein 90, X-associated protein 2, and co-chaperone p23 to form an inactive protein complex (Hankinson, 1995). After ligand binding, the protein complex undergoes a conformational change to expose the nuclear localization sequence of AHR. Then, the AHR-ligand protein complex translocates into the nucleus to act as a transcription factor. In the nucleus, AHR binds to aryl hydrocarbon receptor nuclear translocator (ARNT) to promote transcription of a wide variety of genes, such as cytochrome P450 (CYP) family, AHR repressor, et al. (Wright et al., 2017; Kou and Dai, 2021). In the present study, we aimed to investigate the effects of JPYSF on tryptophan metabolism and AHR signaling activation in adenine-induced CKD rat model.

## Materials and Methods

### JPYSF Extract and Antibodies

JPYSF consists of 8 herbs, namely Astragalus mongholicus Bunge [Fabaceae] (30 g), *Atractylodes macrocephala* Koidz. [Asteraceae] (10 g), *Dioscorea oppositifolia* L. [Dioscoreaceae] (30 g), *Cistanche deserticola* Ma [Orobanchaceae] (10 g), *Wurfbainia vera* (Blackw.) Skornick. & A.D. Poulsen [Zingiberaceae] (10 g), *Salvia miltiorrhiza* Bunge [Lamiaceae] (15 g), *Rheum palmatum* L. [Polygonaceae] (10 g), and *Glycyrrhiza uralensis* Fisch. ex DC. [Fabaceae] (6 g). The plant names were validated using http://mpns.kew.org/mpns-portal/?_ga=1.111763972.1427522246.1459077346. The preparation and quality control of JPYSF extract have been described in detail in our previous studies (Liu et al., 2018; Wang F. et al., 2020). The multiple reaction monitoring (MRM) chromatograms of 9 main compounds present in JPYSF extract were shown in Supplementary Figure S1. The primary antibodies used in the present study included rabbit anti-fibronectin (FN), rabbit anti-type IV collagen (Col-IV), rabbit anti-CYP1B1 (abcam, Cambridge, MA, United States), mouse anti-α-smooth muscle actin (α-SMA), mouse anti-β-actin (Sigma-Aldrich, St Louis, MO, United States), rabbit anti-AHR, rabbit anti-CYP1A2, mouse anti-glyceraldehyde-3-phosphate dehydrogenase (GAPDH) (Proteintech, Wuhan, China), rabbit anti-ARNT (Cell Signaling Technology, Beverly, MA, United States), and rabbit anti-CYP1A1 (Bioss, Beijing, China). Horseradish peroxidase (HRP)-conjugated secondary antibodies were purchased from Cell Signaling Technology (Beverly, MA, United States).

### Animals and Treatment

Healthy male Sprague-Dawley (SD) rats (6–8 weeks, 150–180 g) were provided by Guangdong Medical Laboratory Animal Center (Foshan, China). All rats were acclimated for one week with free access to food and water. A total of 18 rats were randomly divided into three groups: the control group (*n* = 6); the CKD group (*n* = 6); and the JPYSF treatment group (CKD + JPYSF, *n* = 6). CKD was induced by feeding a diet containing 0.75% adenine for 4 weeks as our previous reported (Liu et al., 2019). Rats in the CKD + JPYSF group were given 10.89 g/kg JPYSF per day by gavage for 4 weeks starting from the 3rd week of the adenine-containing diet. Rats in the control and CKD groups were gavaged with equal amounts of double-distilled water. At the end of experiment, rats were euthanized and blood and kidney samples were collected rapidly for further analysis. The above animal experimental protocol was approved by the Experimental Animal Ethics Committee of Guangzhou University of Chinese Medicine.

### Renal Function Indexes

The evaluation of serum creatinine (Scr) and blood urea nitrogen (BUN) were measured by Creatinine Serum Detection Kit and Blood Urea Nitrogen Detection Kit (StressMarq Biosciences, British Columbia, Canada), respectively.

### Pathology

Paraffin-embedded kidney tissues were cut into 4 µm sections. After deparaffinization and rehydration, kidney sections were stained with periodic acid-Schiff (PAS) and Masson’s trichrome, respectively. Then, representative pictures were captured by using Axio Imager M2 microscope and ZEN 2.6 software (Carl Zeiss, Jena, Germany). Tubular injury was scored according to the atrophy and shedding of tubular epithelial cells and tubular dilation in PAS staining. The scoring rules were as follows: 0 = no tubular injury; 1 = < 10%; 2 = 10%–25%; 3 = 26%–50%; 4 = 51%–75%; and 5 = > 75% tubular injury (Cortes et al., 2018). Quantitative analysis of tubulointerstitial fibrosis in Masson staining was performed using ImageJ software (NIH, Bethesda, MD, United States).

### Western Blotting

Frozen kidney cortexes were pulverized in liquid nitrogen and homogenized in RIPA lysis buffer (Cell Signaling Technology, Beverly, MA, United States) containing a protease inhibitor cocktail. Equal amounts of proteins were separated on 7% or 10% SDS-PAGE gels and then transferred to nitrocellulose membranes (Millipore, Billerica, MA, United States). The membranes were blocked with 5% skim milk for 1 h at room temperature and incubated with primary antibodies overnight at 4°C. The next day the blots were incubated with the secondary antibodies for 1 h at room temperature and then were visualized and quantified by ChemiDoc MP imaging system and Image Lab software version 5.1 (Bio-Rad Laboratories, Hercules, CA, United States).

### Immunohistochemistry

Paraffin-embedded kidney tissues were cut into 6 μm sections. After dewaxing and antigen repair, the slides were incubated with 3% hydrogen peroxide for 10 min and were blocked with 10% goat serum for 1 h at room temperature. Then, the primary antibodies were added to the sections and incubated overnight at 4°C. The following day, the sections were treated with the corresponding rabbit or mouse SignalStain Boost Detection Reagent (Cell Signaling Technology) for 30 min and then developed with SignalStain diaminobenzidine (DAB) substrate (Cell Signaling Technology) to produce a brown product. The integrated optical density (IOD) values of the positively stained areas were measured using ImageJ software (NIH, Bethesda, MD, United States). Two microscopic fields (×200) of each rat and three rats in each group were measured in a blinded manner.

### Metabolites Extraction

Serum samples were obtained from fully clotted blood by centrifugation at 2,000 rpm for 10 min. A 100 μl aliquot of each individual sample was precisely transferred to an Eppendorf tube. After the addition of 400 μl of extract solution (methanol:acetonitrile = 1:1, precooled at −40°C, containing 0.1% formic acid and isotopically-labelled internal standard mixture), the samples were vortexed for 30 s and sonicated for 5 min in the ice-water bath, followed by subsiding at −40°C for 1 h. After centrifugation (15 min, 12,000 rpm, and 4°C), a 400 μl aliquot of the supernatant was transferred to an Eppendorf tube. Then the supernatant was evaporated to dryness under a gentle stream of nitrogen and was reconstituted in 100 μl water containing 0.1% formic acid. After centrifugation (15 min, 12,000 rpm, and 4°C), the clear supernatant was subjected to UHPLC-MS/MS analysis.

### Standard Solution Preparation

A total of 28 tryptophan metabolites were quantified in the present study (Supplementary Table S1). Stock solutions were individually prepared by dissolving or diluting each standard substance to give a final concentration of 1 mmol/L. An aliquot of each of the stock solutions was transferred to an Eppendorf tube form a mixed working standard solution. A series of calibration standard solutions were then prepared by stepwise dilution of this mixed standard solution (containing isotopically-labelled internal standard mixture in identical concentrations with the samples).

### UHPLC-MS/MS Analysis

The UHPLC separation was carried out using an EXIONLC System (Sciex), equipped with a Waters ACQUITY UPLC HSS T3 column (100 × 2.1 mm, 1.8 μm, Waters). The mobile phase A was 0.1% formic acid in water, and the mobile phase B was 0.1% formic acid in acetonitrile. The column temperature was set at 40°C. The auto-sampler temperature was set at 4°C and the injection volume was 5 μl. A SCIEX 6500 QTRAP + triple quadrupole mass spectrometer (Sciex) equipped with an IonDrive Turbo V electrospray ionization (ESI) interface was applied for assay development. Typical ion source parameters were: Curtain Gas = 40 psi, IonSpray Voltage = ±4500 V, temperature = 500°C, Ion Source Gas 1 = 30 psi, Ion Source Gas 2 = 30 psi. The multiple reaction monitoring (MRM) parameters for each of the targeted analytes were optimized using flow injection analysis, by injecting the standard solutions of the individual analytes into the API source of the mass spectrometer. Several most sensitive transitions were used in the MRM scan mode to optimize the collision energy for each Q1/Q3 pair (Supplementary Table S2). Among the optimized MRM transitions per analyte, the Q1/Q3 pairs that showed the highest sensitivity and selectivity were selected as ‘quantifier’ for quantitative monitoring. The additional transitions acted as ‘qualifier’ for the purpose of verifying the identity of the target analytes. SCIEX Analyst Work Station software (Version 1.6.3) and Sciex MultiQuant software (Version 3.0.3) were employed for MRM data acquisition and processing.

### Calibration Curves

Calibration solutions were subjected to UHPLC-MS/MS analysis using the methods described above. In calibration curves, y is the ratio of peak areas for analyte/IS, and x is the ratio of concentration for analyte/IS. Least squares method was used for the regression fitting. 1/x weighting was applied in the curve fitting since it provided highest accuracy and correlation coefficient (R^2^). The level was excluded from the calibration if the accuracy of calibration was not within 80%–120%.

### Limit of Detection and Limit of Quantitation

The calibration standard solution was diluted stepwise, with a dilution factor of 2. These standard solutions were subjected to UHPLC-MS/MS analysis. The signal-to-noise ratios (S/N) were used to determine the lower limits of detection (LLODs) and lower limits of quantitation (LLOQs). The LLODs and LLOQs were defined as the analyte concentrations that led to peaks with signal-to-noise ratios (S/N) of 3 and 10, respectively, according to the US FDA guideline for bioanalytical method validation.

### Precision and Accuracy

The precision of the quantitation was measured as the relative standard deviation (RSD), determined by injecting analytical replicates of a QC sample. The accuracy of quantitation was measured as the analytical recovery of the QC sample determined. The percent recovery was calculated as [(mean observed concentration)/(spiked concentration)] × 100%.

### Statistical Analysis

Data in the study were expressed as the mean ± standard error of the mean (SEM). One-way ANOVA followed by Tukey’s post hoc test or Student’s t-test was applied for calculating statistical differences. The statistical analyses and graphs were performed by using GraphPad Prism 9 Software (La Jolla, CA, United States). *p* < 0.05 was considered statistically significant differences.

## Results

### JPYSF Improved Renal Function and Pathological Injury in CKD Rats

**FIGURE 1:**
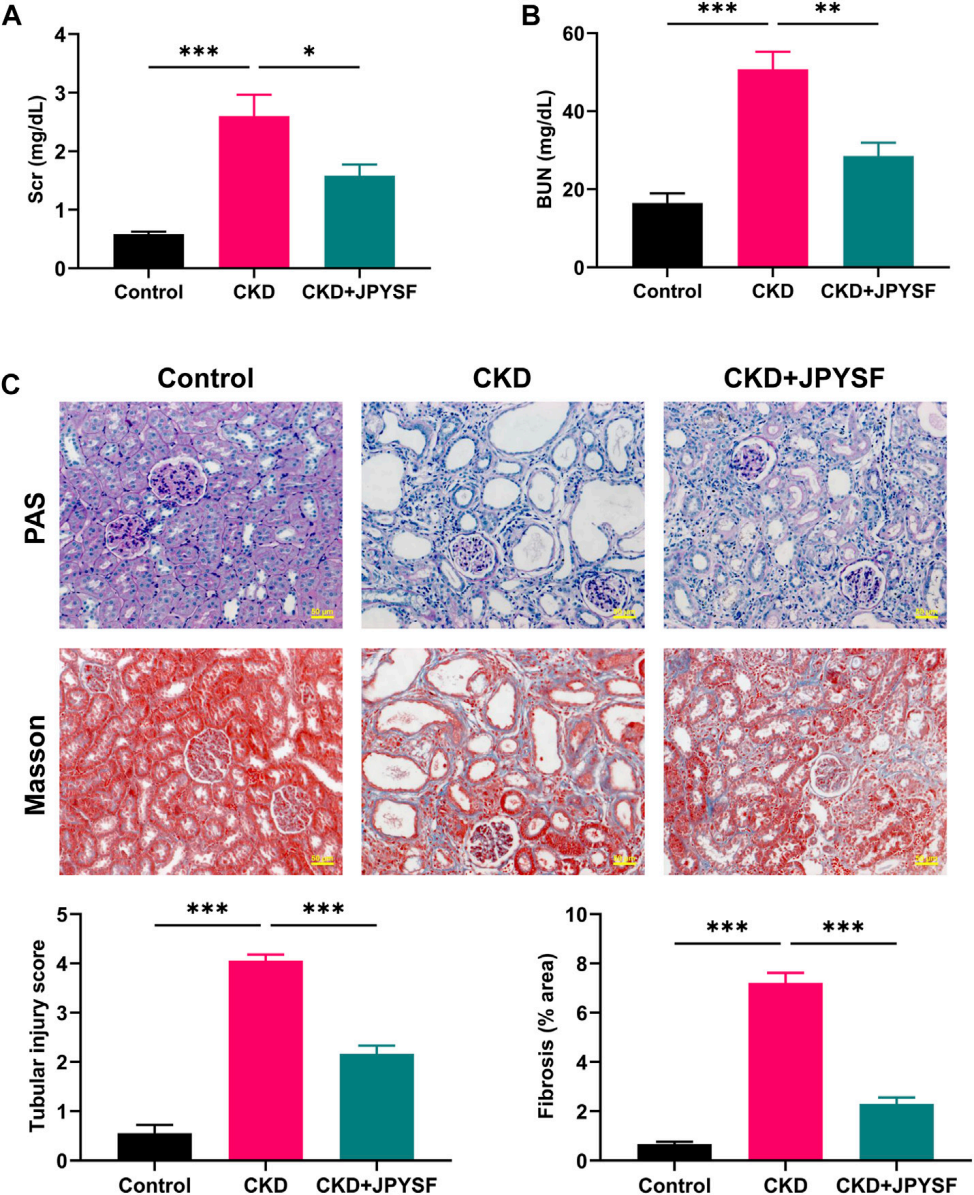
Effects of JPYSF on renal function and pathological injury in adenine-induced CKD rats. **(A)** Serum creatinine levels. **(B)** Blood urea nitrogen levels. **(C)** Representative PAS and Masson staining images and quantitative analysis of tubular injury and fibrosis. All images are shown at identical magnification, ×200, scale bar = 50 μm. Values are expressed as mean ± SEM, *n* = 6 rats per group (*p < 0.05, **p < 0.01, and ***p < 0.001 between the indicated two groups).

Compared with the control group, the levels of Scr (2.60 ± 0.37 mg/dl *vs.* 0.58 ± 0.04 mg/dl, *p* < 0.001) and BUN (50.79 ± 4.49 mg/dl *vs.* 16.51 ± 2.47 mg/dl, *p* < 0.001) were significantly increased in CKD rats (Figures 1A,B). JPYSF treatment remarkably reduced the levels of Scr and BUN in CKD rats. In PAS staining, CKD rats showed shedding of renal tubular epithelial cells and tubular dilation. Masson staining showed obvious collagen deposition in the tubulointerstitium of CKD rats. These pathological injuries were ameliorated by JPYSF treatment (Figure 1C). In addition, administration of JPYSF had no apparent effect on liver function as evaluated by serum levels of alanine transaminase (ALT) and aspartate transaminase (AST) (Supplementary Figure S2). These results indicated that JPYSF could protect against adenine-induced CKD without side effect on liver function.

### JPYSF Alleviated Renal Fibrosis in CKD Rats

**FIGURE 2:**
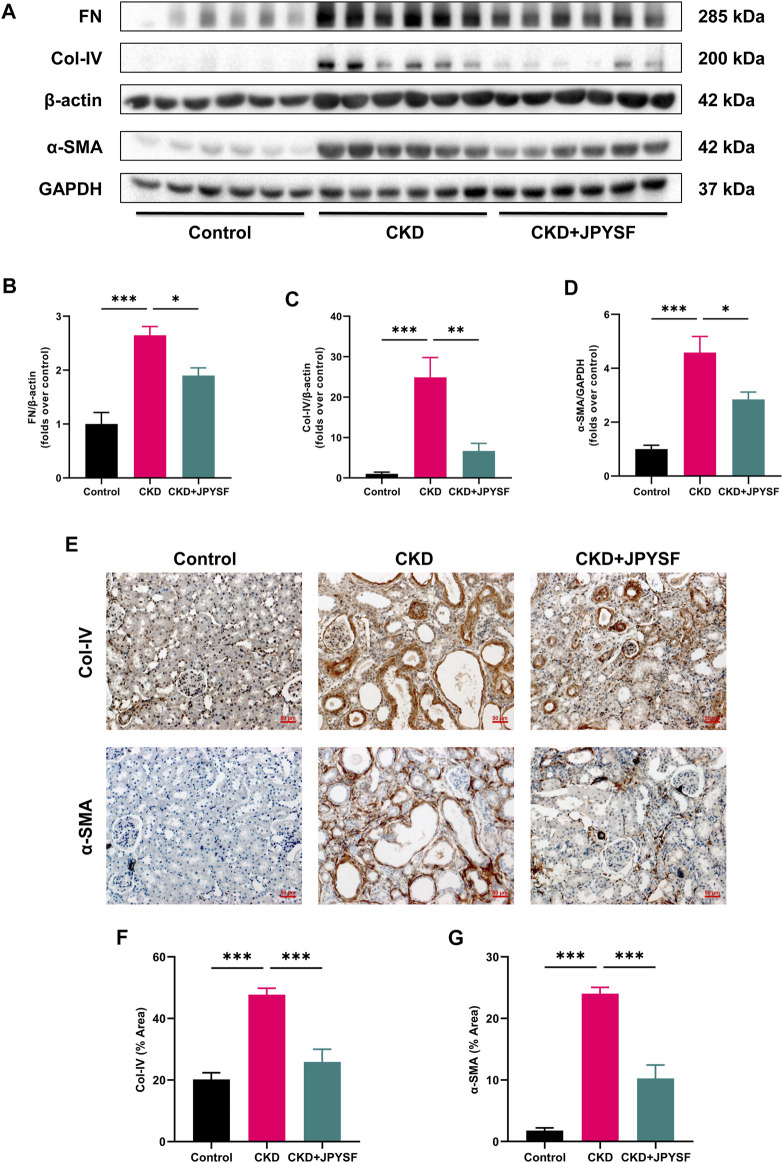
Effects of JPYSF on renal fibrosis in adenine-induced CKD rats. **(A)** Representative Western blot images of FN, Col-IV, and α-SMA expression in the kidneys of rats. **(B–D)** Densitometric analysis of FN, Col-IV, and α-SMA normalized to β-actin or GAPDH content (*n* = 6). **(E)** Representative immunohistochemistry images of Col-IV and α-SMA expression in the kidneys of rats. All images are shown at identical magnification, ×200, scale bar = 50 μm. **(F,G)** Quantitative analysis of positive staining areas for Col-IV and α-SMA (*n* = 3). Values are expressed as mean ± SEM (*p < 0.05, **p < 0.01, and ***p < 0.001 between the indicated two groups).

FN, Col-IV, and α-SMA are indicators of renal fibrosis. In Western blot analysis, the expression of these three indicators in the kidneys of CKD rats was significantly increased (*p* < 0.001), and could be reversed by JPYSF treatment (*p* < 0.05) (Figures 2A–D). Further results from immunohistochemistry showed that JPYSF reduced the elevated expression of Col-IV and α-SMA in the kidneys of CKD rats (*p* < 0.001, Figures 2E–G). These data suggested that JPYSF attenuated renal fibrosis in adenine-induced CKD.

### Accuracy of the UHPLC-MS/MS Method

As shown in Supplementary Table S3, the LLODs ranged from 0.01 to 312.50 nmol/L, and the LLOQs ranged from 0.02 to 625.00 nmol/L for the targeted metabolites. Correlation coefficients (R^2^) of regression fitting were above 0.9961 for all the analytes, indicating a good quantitative relationship between the MS responses and the analyte concentrations, which was satisfying for targeted metabolomics analysis. Supplementary Table S4 lists the analytical recoveries and RSDs of the QC samples. The recoveries determined were 86.05%–106.62% for all the analytes, with all RSDs below 12.32%. These analysis metrics indicated that the method allowed accurate quantitation of the targeted metabolites in the biological sample in the concentration range described as above.

### Comparison of Metabolic Profiles Between Groups

**FIGURE 3:**
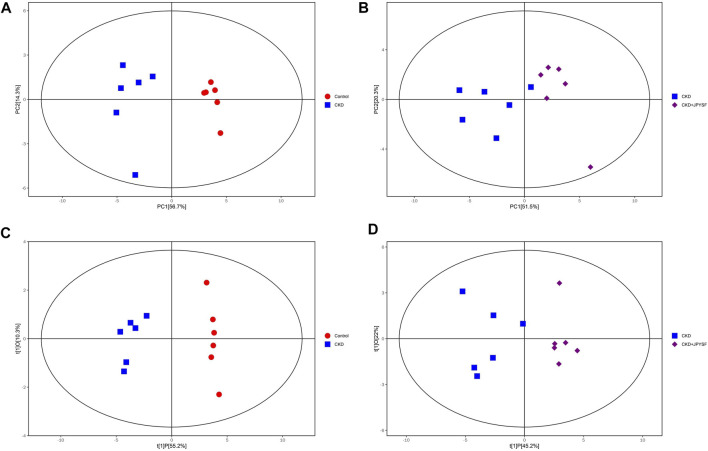
The score scatter plots of PCA and OPLS-DA model. **(A)** PCA score plot of the control group versus the CKD group. **(B)** PCA score plot of the CKD group versus the CKD + JPYSF group. **(C)** OPLS-DA score plot of the control group versus the CKD group. **(D)** OPLS-DA score plot of the CKD group versus the CKD + JPYSF group. The red circle represents the control group, the blue square represents the CKD group, and the purple diamond represents the CKD + JPYSF group.

To compare the differences in metabolic profiles between groups, principal component analysis (PCA) and orthogonal projections to latent structures-discriminant analysis (OPLS-DA) were performed. The PCA score plots showed clear separation when comparing the control group with the CKD group, but not complete separation when comparing the CKD group with the CKD + JPYSF group (Figures 3A,B). In the more reliable OPLS-DA model, the score plots could all be divided into two distinct clusters in the above two sets of comparisons (Figures 3C,D). These results suggested that metabolic profiles were altered in the CKD model and could be modulated by JPYSF treatment.

### Comparison of the Changing Trends of Tryptophan Metabolites Between Groups

**FIGURE 4:**
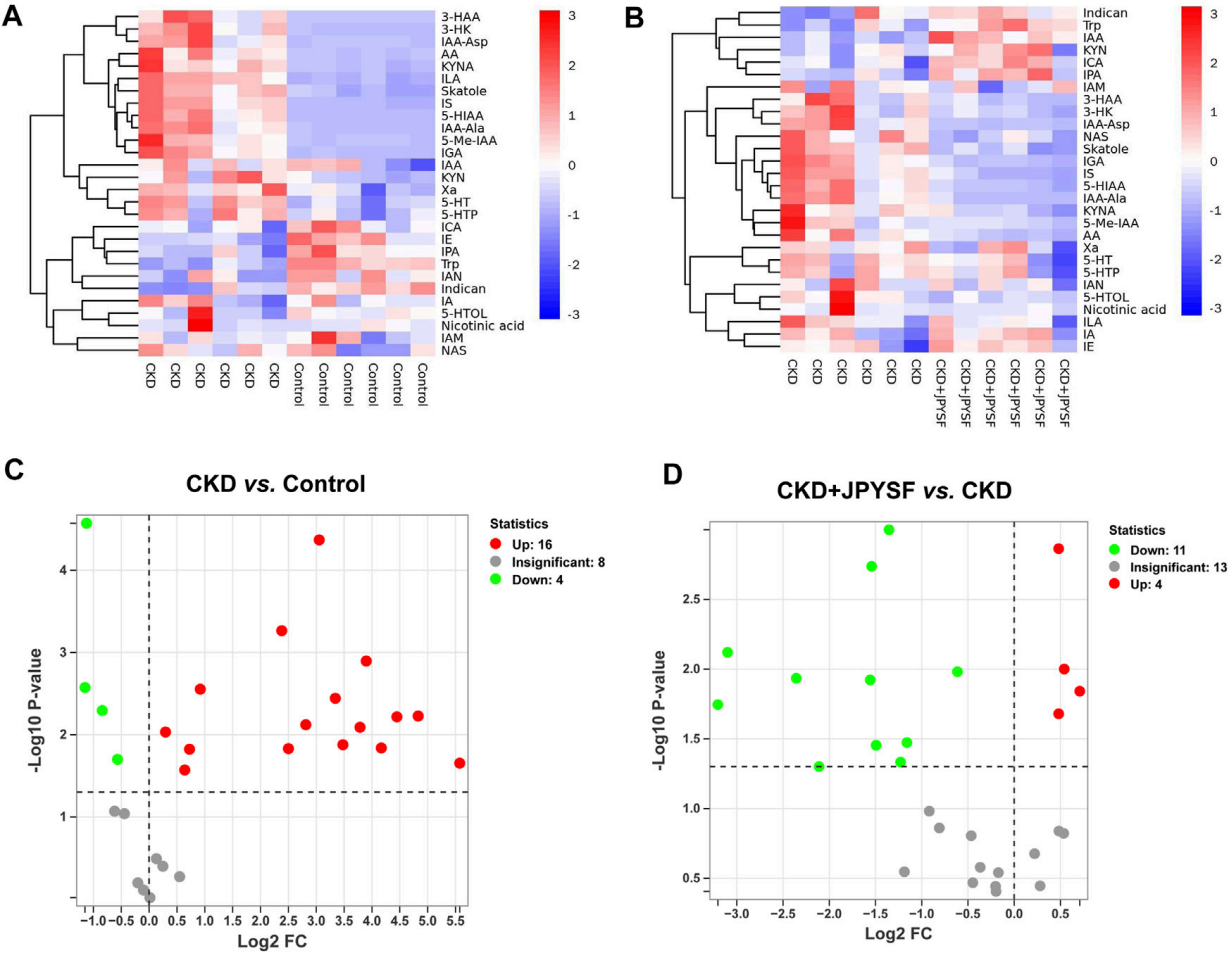
Differences in tryptophan metabolites content in different groups. **(A)** Heatmap analysis between the control group and the CKD group. **(B)** Heatmap analysis between the CKD group and the CKD + JPYSF group. In the heatmap, red represents increase and blue represents decrease. **(C)** Volcano plot analysis of the CKD group compared with the control group. **(D)** Volcano plot analysis of the CKD + JPYSF group compared with the CKD group. In the volcano plot, red represents significant up-regulation, green represents significant down-regulation, and grey represents insignificant difference.

A total of 28 metabolites of tryptophan were detected in this study. The changes in the content of each metabolite in the comparison of the control group with the CKD group and the comparison of the CKD group with the CKD + JPYSF group were summarized in Figures 4A,B. Compared with the control group, the levels of 16 metabolites were significantly increased and the levels of 4 metabolites were significantly decreased in the serum of rats in the CKD group (Figure 4C). These 16 up-regulated tryptophan metabolites had good diagnostic value for CKD with area under the curve (AUC) at 0.833–1.000 in receiver operating characteristic (ROC) analysis (Supplementary Figure S3). Administration of JPYSF treatment significantly down-regulated 11 metabolites and up-regulated 4 metabolites contents in CKD rats (Figure 4D). These results indicated that JPYSF could regulate tryptophan metabolism in CKD rats.

### Identification of Tryptophan Metabolites Modulated by JPYSF in CKD Rats

**FIGURE 5:**
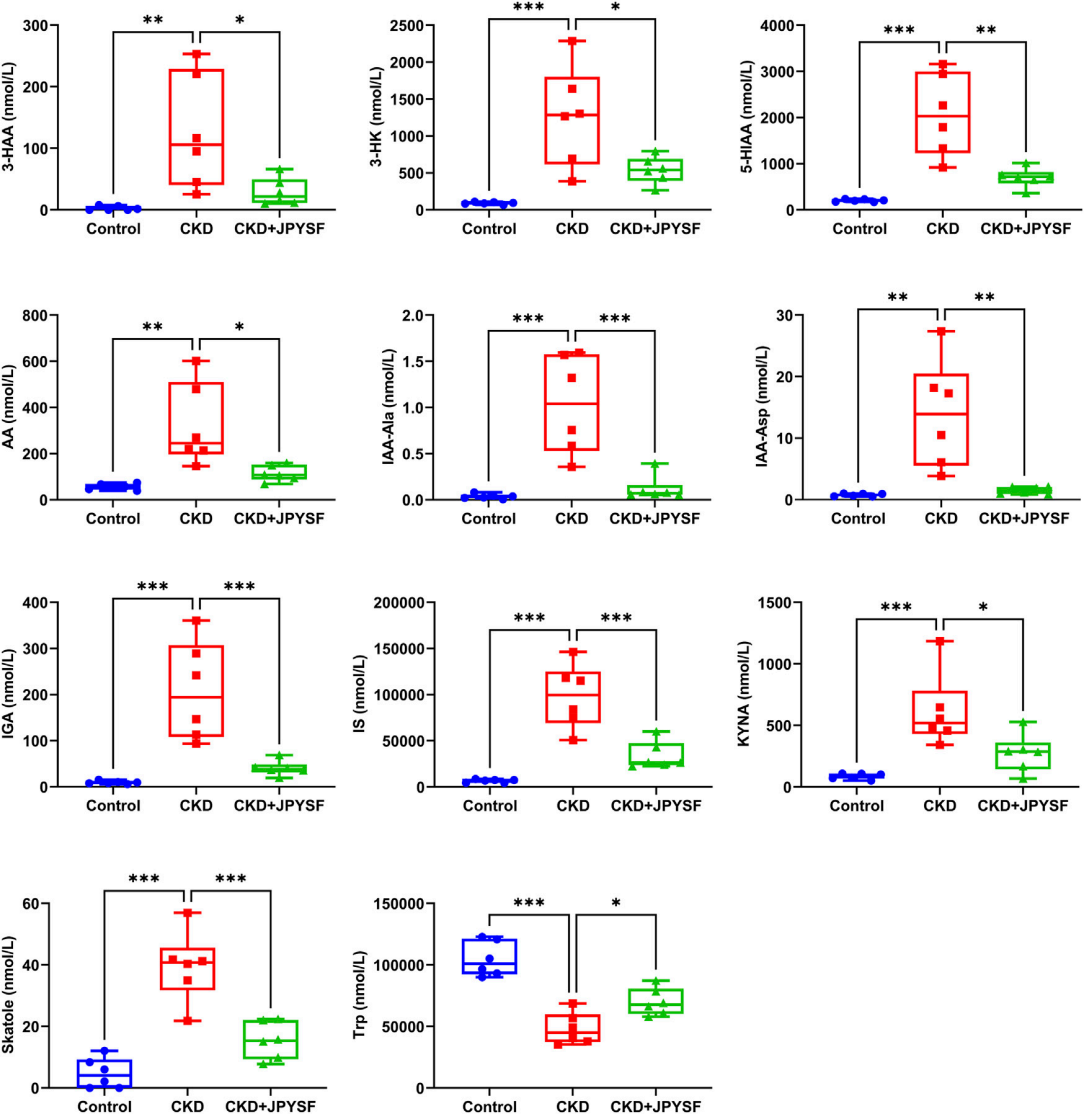
Effects of JPYSF on 11 tryptophan metabolites in adenine-induced CKD rats. Values are expressed as mean ± SEM, *n* = 6 rats per group (*p < 0.05, **p < 0.01, and ***p < 0.001 between the indicated two groups).

It was found that JPYSF could significantly restore the levels of 11 tryptophan metabolites by comparing the data of the three groups. Among these metabolites, 3-hydroxyanthranilic acid (3-HAA), 3-hydroxykynurenine (3-HK), 5-hydroxyindoleacetic acid (5-HIAA), anthranilic acid (AA), indole-3-acetyl-alanine (IAA-Ala), indole-3-acetyl-aspartate (IAA-Asp), 3-indoleglyoxylic acid (IGA), indoxylsulfate (IS), kynurenic acid (KYNA), and skatole were significantly elevated in the CKD group and were significantly down-regulated by JPYSF treatment. The level of L-tryptophan (Trp) was decreased in the CKD group and could be restored by JPYSF (*p* < 0.05, Figure 5).

### JPYSF Suppressed AHR Signaling Activation in CKD Rats

**FIGURE 6:**
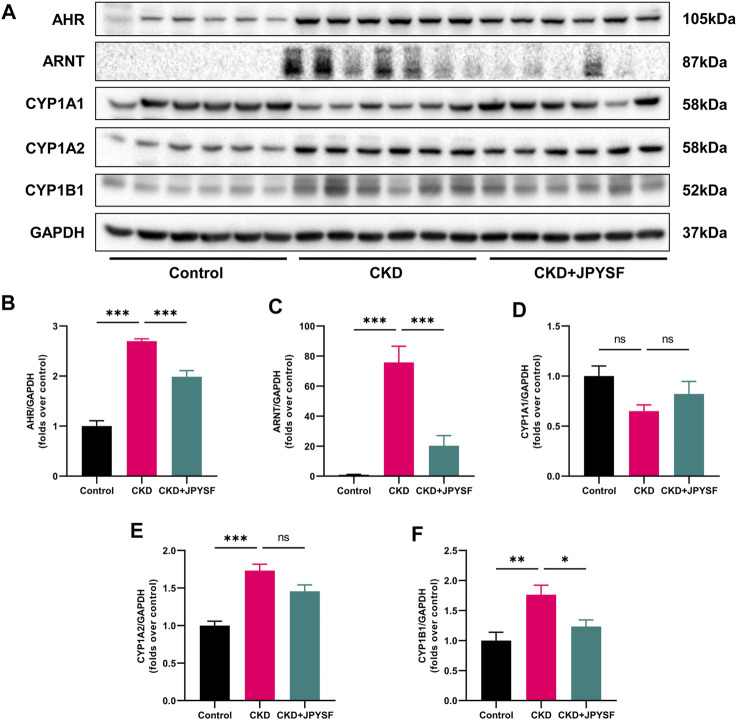
Effects of JPYSF on AHR signaling in adenine-induced CKD rats. **(A)** Representative Western blot images of AHR, ARNT, CYP1A1, CYP1A2, and CYP1B1 expression in the kidneys of rats. **(B–F)** Densitometric analysis of AHR, ARNT, CYP1A1, CYP1A2, and CYP1B1 normalized to GAPDH content. Values are expressed as mean ± SEM, *n* = 6 rats per group (*p < 0.05, **p < 0.01, and ***p < 0.001 between the indicated two groups, ns means not significant).

Tryptophan metabolites are important endogenous AHR ligands in CKD (Brito et al., 2017). We further examined the activation of AHR signaling in the kidneys of CKD rats with or with JPYSF. As shown in Figure 6, the expression of AHR and ARNT were significantly increased in the kidneys of CKD rats, which was suppressed by JPYSF treatment (*p* < 0.001). The downstream proteins of AHR signaling, CYP1A2 and CYP1B1, were significantly up-regulated in the CKD group. JPYSF could down-regulate CYP1B1 expression in CKD rats (*p* < 0.05, Figure 6). These results suggested that AHR signaling was activated in the kidney of CKD rat and could be partially restored by JPYSF treatment.

## Discussion

In the present study, we investigated the effects of JPYSF on tryptophan metabolism and AHR signaling activation in adenine-induced CKD rat model. The results showed that JPYSF alleviated adenine-induced kidney dysfunction and renal fibrosis. JPYSF could modulate tryptophan metabolism, reduce the levels of tryptophan-derived uremic toxins, and inhibit AHR signaling activation. These results provided insights into the molecular mechanism of JPYSF in adenine-induced CKD.

Tryptophan (Trp) is an essential amino acid and plays a crucial role in protein biosynthesis and diverse metabolic functions (Kałużna-Czaplińska et al., 2019). There are three metabolic pathways for tryptophan: 1) The kynurenine pathway metabolizes 95% of tryptophan. This pathway is started by the rate-limiting enzymes tryptophan 2,3-dioxygenase (TDO) and indoleamine 2,3-dioxygenase (IDO) that catalyze the production of kynurenine from tryptophan. Then, various metabolites are derived from kynurenine, such as 3-HAA, 3-HK, KYNA, AA, and quinolinic acid (QA) (Fatokun et al., 2013). It has been reported that the serum level of Trp was decreased whereas metabolites of the kynurenine pathway were increased in CKD patients (Saito et al., 2000; Pawlak et al., 2001). Similar with this, our results also showed a decreased level of Trp and increased levels of 3-HAA, 3-HK, AA, and KYNA in the serum of adenine-induced CKD rat. Importantly, JPYSF treatment significantly reduced the levels of kynurenine metabolites and restored Trp content in CKD rat, which reflected its regulatory effect on the kynurenine pathway. 2) The serotonin pathway that leads to melatonin. 3) The indolic pathway that leads to the indolic uremic toxins. In this pathway, tryptophan is converted to indole by the gut microbiota and absorbed into the blood circulation (Aronov et al., 2011). In the liver, indoles are metabolized to IS and indoxyl-β-D glucuronide (IDG) (Meyer and Hostetter, 2012). IS, IAA, and IDG are commonly accumulated in patients with CKD (Duranton et al., 2012). In the present study, JPYSF markedly lowered the elevated levels of IS, IAA-Ala, IAA-Asp, and IGA in CKD rats, suggesting an inhibitory effect on the indolic pathway.

The AHR is a ligand-activated transcription factor that was originally discovered to mediate the toxicity 2,3,7,8-tetrachlorodibenzo-p-dioxin (TCDD) (Poland and Knutson, 1982). In recent years, however, it has become apparent that AHR is activated not only by exogenous toxins but also by endogenous ligands (Safe et al., 2020). Patients with CKD are exposed to uremic toxins, especially those derived from tryptophan metabolism. Dou et al. reported that compared to sera from healthy controls, sera from CKD patients displayed a strong AHR-activating potential; strongly correlated with estimated glomerular filtration rate (eGFR) and with the IS concentration. In mice with 5/6 nephrectomy, there was also an increased serum AHR-activating potential and could be enhanced by IS injection (Dou et al., 2018). AHR transactivating activity was higher in patients with pre-dialysis CKD stage IV or V than in patients undergoing dialysis, which may be related to the concentration of uremic toxins (Kim et al., 2020). The mechanisms of AHR affecting CKD are thought to be related to the crosstalk between AHR signal and rennin angiotensin aldosterone system, transforming growth factor-β1 (TGF-β1) pathway, and nuclear factor kappa B (NF-κB) pathway (Curran and Kopp, 2022). Therefore, AHR signaling may be a pharmacological target in CKD (Mo et al., 2020). Wang et al. identified novel poricoic acids from Poria cocos and found that they attenuated renal fibrosis through the modulation of AHR signaling pathway in TGF-β1-induced cultured human kidney proximal tubular epithelial cells and mice with unilateral ureteral obstruction (Wang M. et al., 2020). Fisetin, a bioactive flavonol, was reported to improve hyperuricemia-induced CKD via regulating gut microbiota-mediated tryptophan metabolism and AHR activation (Ren et al., 2021). In our study, JPYSF was found to suppress the activation of AHR signaling in the kidney of CKD rat. However, the specific compounds targeting AHR signaling in JPYSF require further investigation.

Based on the theory of traditional Chinese medicine, “deficiency in Ben and excess in Biao” is the basic pathogenesis of CKD. “Deficiency in Ben” is mainly due to deficiency of the spleen and kidney, and “excess in Biao” mainly refers to blood stasis, water-dampness and toxins. In JPYSF, Huang-qi, Bai-zhu, Shan-yao, and Rou-cong-rong nourish the spleen and kidney; Dan-shen activates blood circulation, Dou-kou removes dampness, and Da-huang clears toxins. Therefore, the combination of these herbs hits the core pathogenesis of CKD and exerts therapeutic effect. Tryptophan metabolites are one of the sources of uremic toxins in CKD patients. Our data suggested that JPYSF reduced serum tryptophan metabolite levels in CKD rats. This provides an experimental basis for the application of traditional Chinese medicine formula in the treatment of CKD.

## Conclusion

In conclusion, our data demonstrated that JPYSF treatment alleviated renal dysfunction and fibrosis in rat of adenine-induced CKD. This therapeutic effect may be related to the regulation of tryptophan metabolism and inhibition of AHR signaling activation.

## Data Availability

The original contributions presented in the study are included in the article/Supplementary Material, further inquiries can be directed to the corresponding authors.

## References

[B1] Aronov P. A. Luo F. J. Plummer N. S. Quan Z. Holmes S. Hostetter T. H. (2011). Colonic Contribution to Uremic Solutes. J. Am. Soc. Nephrol. 22, 1769–1776. 10.1681/ASN.2010121220 21784895 PMC3171947

[B2] Bikbov B. Purcell C. Levey A. Smith M. Abdoli A. Abebe M. (2020). Global, Regional, and National Burden of Chronic Kidney Disease, 1990-2017: a Systematic Analysis for the Global Burden of Disease Study 2017. Lancet 395, 709–733. 10.1016/S0140-6736(20)30045-3 32061315 PMC7049905

[B3] Brito J. S. Borges N. A. Esgalhado M. Magliano D. C. Soulage C. O. Mafra D. (2017). Aryl Hydrocarbon Receptor Activation in Chronic Kidney Disease: Role of Uremic Toxins. Nephron 137, 1–7. 10.1159/000476074 28490014

[B4] Cortes A. L. Gonsalez S. R. Rioja L. S. Oliveira S. S. C. Santos A. L. S. Prieto M. C. (2018). Protective Outcomes of Low-Dose Doxycycline on Renal Function of Wistar Rats Subjected to Acute Ischemia/reperfusion Injury. Biochim. Biophys. Acta Mol. Basis Dis. 1864, 102–114. 10.1016/j.bbadis.2017.10.005 28987762 PMC5705293

[B5] Curran C. S. Kopp J. B. (2022). Aryl Hydrocarbon Receptor Mechanisms Affecting Chronic Kidney Disease. Front. Pharmacol. 13, 782199. 10.3389/fphar.2022.782199 35237156 PMC8882872

[B6] Dou L. Poitevin S. Sallée M. Addi T. Gondouin B. Mckay N. (2018). Aryl Hydrocarbon Receptor Is Activated in Patients and Mice with Chronic Kidney Disease. Kidney Int. 93, 986–999. 10.1016/j.kint.2017.11.010 29395338

[B7] Duranton F. Cohen G. De Smet R. Rodriguez M. Jankowski J. Vanholder R. (2012). Normal and Pathologic Concentrations of Uremic Toxins. J. Am. Soc. Nephrol. 23, 1258–1270. 10.1681/ASN.2011121175 22626821 PMC3380651

[B8] Fatokun A. A. Hunt N. H. Ball H. J. (2013). Indoleamine 2,3-dioxygenase 2 (Ido2) and the Kynurenine Pathway: Characteristics and Potential Roles in Health and Disease. Amino Acids 45, 1319–1329. 10.1007/s00726-013-1602-1 24105077

[B9] Hankinson O. (1995). The Aryl Hydrocarbon Receptor Complex. Annu. Rev. Pharmacol. Toxicol. 35, 307–340. 10.1146/annurev.pa.35.040195.001515 7598497

[B10] Kalantar-Zadeh K. Jafar T. H. Nitsch D. Neuen B. L. Perkovic V. (2021). Chronic Kidney Disease. Lancet 398, 786–802. 10.1016/S0140-6736(21)00519-5 34175022

[B11] Kałużna-Czaplińska J. Gątarek P. Chirumbolo S. Chartrand M. S. Bjørklund G. (2019). How Important Is Tryptophan in Human Health? Crit. Rev. Food Sci. Nutr. 59, 72–88. 10.1080/10408398.2017.1357534 28799778

[B12] Kim J. T. Kim S. H. Min H. K. Jeon S. J. Sung S. A. Park W. H. (2020). Effect of Dialysis on Aryl Hydrocarbon Receptor Transactivating Activity in Patients with Chronic Kidney Disease. Yonsei Med. J. 61, 56–63. 10.3349/ymj.2020.61.1.56 31887800 PMC6938787

[B13] Kou Z. Dai W. (2021). Aryl Hydrocarbon Receptor: Its Roles in Physiology. Biochem. Pharmacol. 185, 114428. 10.1016/j.bcp.2021.114428 33515530 PMC8862184

[B14] Li X. Wang H. (2005). Chinese Herbal Medicine in the Treatment of Chronic Kidney Disease. Adv. Chronic Kidney Dis. 12, 276–281. 10.1016/j.ackd.2005.03.007 16010642

[B15] Liu X. Chen J. Liu X. Wang D. Zheng P. Qi A. (2018). Jian-Pi-Yi-Shen Formula Ameliorates Chronic Kidney Disease: Involvement of Mitochondrial Quality Control Network. BMC Complement. Altern. Med. 18, 340. 10.1186/s12906-018-2395-2 30572886 PMC6302435

[B16] Liu X. Deng R. Wei X. Wang Y. Weng J. Lao Y. (2021a). Jian-Pi-Yi-Shen Formula Enhances Perindopril Inhibition of Chronic Kidney Disease Progression by Activation of SIRT3, Modulation of Mitochondrial Dynamics, and Antioxidant Effects. Biosci. Rep. 41, BSR20211598. 10.1042/BSR20211598 34633033 PMC8536834

[B17] Liu X. Zhang B. Huang S. Wang F. Zheng L. Lu J. (2019). Metabolomics Analysis Reveals the Protection Mechanism of Huangqi-Danshen Decoction on Adenine-Induced Chronic Kidney Disease in Rats. Front. Pharmacol. 10, 992. 10.3389/fphar.2019.00992 31551789 PMC6747014

[B18] Liu X. Liu S. Luo D. Huang S. Wang F. Zhang B. (2020). Involvement of Circulating Exosomal MicroRNAs in Jian-Pi-Yi-Shen Formula Protection against Adenine-Induced Chronic Kidney Disease. Front. Pharmacol. 11, 622658. 10.3389/fphar.2020.622658 33603670 PMC7884821

[B19] Liu X. Liu S. Zhang B. Luo D. Huang S. Wang F. (2021b). Jian-Pi-Yi-Shen Formula Alleviates Chronic Kidney Disease in Two Rat Models by Modulating QPRT/NAD(+)/SIRT3/Mitochondrial Dynamics Pathway. Evid. Based Complement. Altern. Med. 2021, 6625345. 10.1155/2021/6625345 PMC8687808 34938344

[B20] Lu J. Liu X. Liao Y. Wang D. Chen J. Li S. (2018). Jian-Pi-Yi-Shen Formula Regulates Inflammatory Cytokines Production in 5/6 Nephrectomized Rats via Suppression of NF-Κb Activation. Evid. Based Complement. Altern. Med. 2018, 7203547. 10.1155/2018/7203547 PMC6077543 30108662

[B21] Meyer T. W. Hostetter T. H. (2012). Uremic Solutes from Colon Microbes. Kidney Int. 81, 949–954. 10.1038/ki.2011.504 22318422

[B22] Mo Y. Lu Z. Wang L. Ji C. Zou C. Liu X. (2020). The Aryl Hydrocarbon Receptor in Chronic Kidney Disease: Friend or Foe? Front. Cell. Dev. Biol. 8, 589752. 10.3389/fcell.2020.589752 33415104 PMC7784643

[B23] Pawlak D. Pawlak K. Malyszko J. Mysliwiec M. Buczko W. (2001). Accumulation of Toxic Products Degradation of Kynurenine in Hemodialyzed Patients. Int. Urol. Nephrol. 33, 399–404. 10.1023/a:1015238418500 12092667

[B24] Poland A. Knutson J. C. (1982). 2,3,7,8-tetrachlorodibenzo-p-dioxin and Related Halogenated Aromatic Hydrocarbons: Examination of the Mechanism of Toxicity. Annu. Rev. Pharmacol. Toxicol. 22, 517–554. 10.1146/annurev.pa.22.040182.002505 6282188

[B25] Ren Q. Cheng L. Guo F. Tao S. Zhang C. Ma L. (2021). Fisetin Improves Hyperuricemia-Induced Chronic Kidney Disease via Regulating Gut Microbiota-Mediated Tryptophan Metabolism and Aryl Hydrocarbon Receptor Activation. J. Agric. Food Chem. 69, 10932–10942. 10.1021/acs.jafc.1c03449 34505780

[B26] Safe S. Jin U. H. Park H. Chapkin R. S. Jayaraman A. (2020). Aryl Hydrocarbon Receptor (AHR) Ligands as Selective AHR Modulators (SAhRMs). Int. J. Mol. Sci. 21, 6654. 10.3390/ijms21186654 PMC7555580 32932962

[B27] Saito K. Fujigaki S. Heyes M. P. Shibata K. Takemura M. Fujii H. (2000). Mechanism of Increases in L-Kynurenine and Quinolinic Acid in Renal Insufficiency. Am. J. Physiol. Ren. Physiol. 279, F565–F572. 10.1152/ajprenal.2000.279.3.F565 10966936

[B28] Walker J. A. Richards S. Belghasem M. E. Arinze N. Yoo S. B. Tashjian J. Y. (2020). Temporal and Tissue-specific Activation of Aryl Hydrocarbon Receptor in Discrete Mouse Models of Kidney Disease. Kidney Int. 97, 538–550. 10.1016/j.kint.2019.09.029 31932072 PMC9721455

[B29] Wang F. Huang S. Chen Q. Hu Z. Li Z. Zheng P. (2020). Chemical Characterisation and Quantification of the Major Constituents in the Chinese Herbal Formula Jian-Pi-Yi-Shen Pill by UPLC-Q-TOF-MS/MS and HPLC-QQQ-MS/MS. Phytochem. Anal. 31, 915–929. 10.1002/pca.2963 32488993

[B30] Wang M. Hu H. H. Chen Y. Y. Chen L. Wu X. Q. Zhao Y. Y. (2020). Novel Poricoic Acids Attenuate Renal Fibrosis through Regulating Redox Signalling and Aryl Hydrocarbon Receptor Activation. Phytomedicine 79, 153323. 10.1016/j.phymed.2020.153323 32920287

[B31] Wright E. J. De Castro K. P. Joshi A. D. Elferink C. J. (2017). Canonical and Non-canonical Aryl Hydrocarbon Receptor Signaling Pathways. Curr. Opin. Toxicol. 2, 87–92. 10.1016/j.cotox.2017.01.001 32296737 PMC7158745

