# Supplementary material for: Jian-Pi-Yi-Shen Formula Improves Adenine-Induced Chronic Kidney Disease via Regulating Tryptophan Metabolism and Aryl Hydrocarbon Receptor Signaling
Source: Front Pharmacol. 2022 Jul 5;13:922707. doi: 10.3389/fphar.2022.922707 (PMC9294467; doi:10.3389/fphar.2022.922707)
Supplement: Supplementary file 5 [file DataSheet1.docx]

**Supplementary Figures**


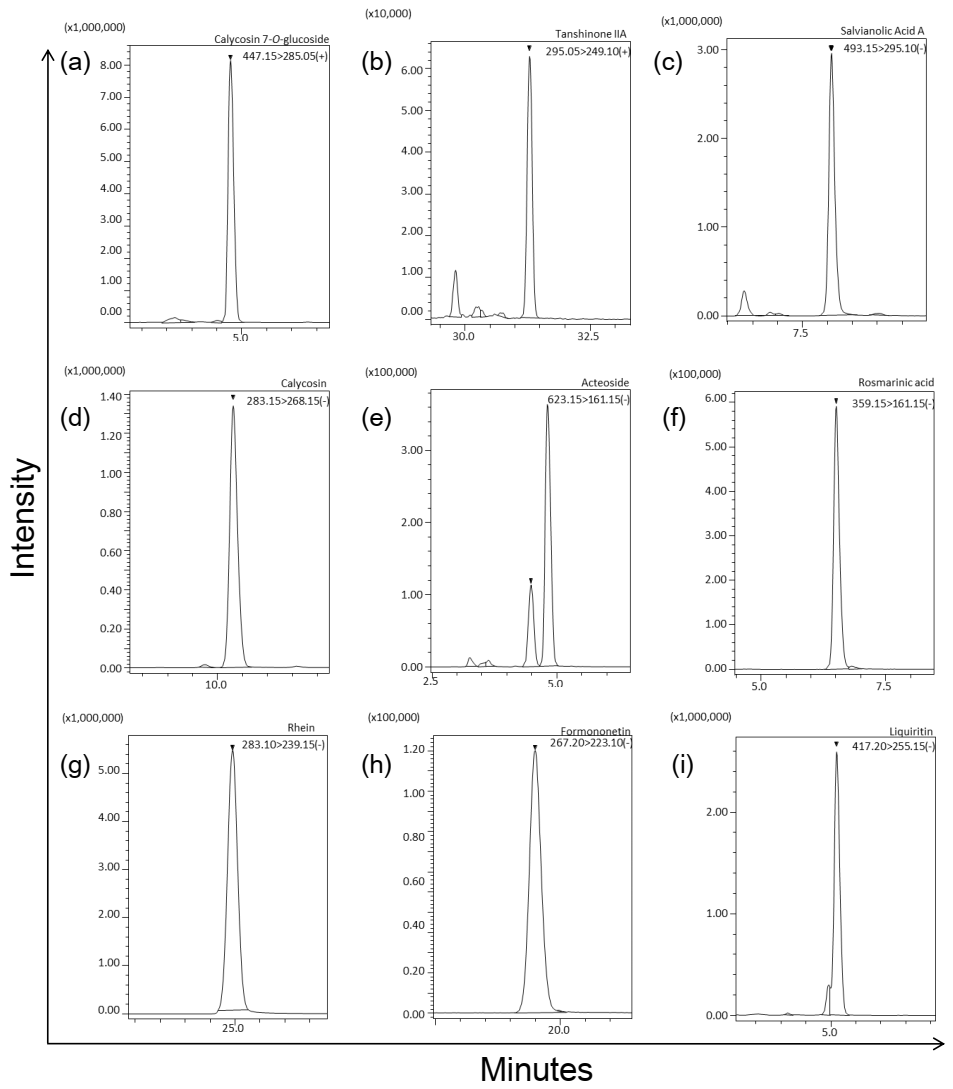


**Supplementary Figure S1.** High-performance liquid chromatography method coupled with triple-quadrupole tandem mass spectrometry (HPLC-QQQ-MS/MS) MRM chromatograms of the JPYSF extract. (a) calycosin 7-O-glucoside; (b) tanshinone IIA; (c) salvianolic acid A; (d) calycosin; (e) acteoside; (f) rosmarinic acid; (g) rhein; (h) formononetin; (i) liquiritin.


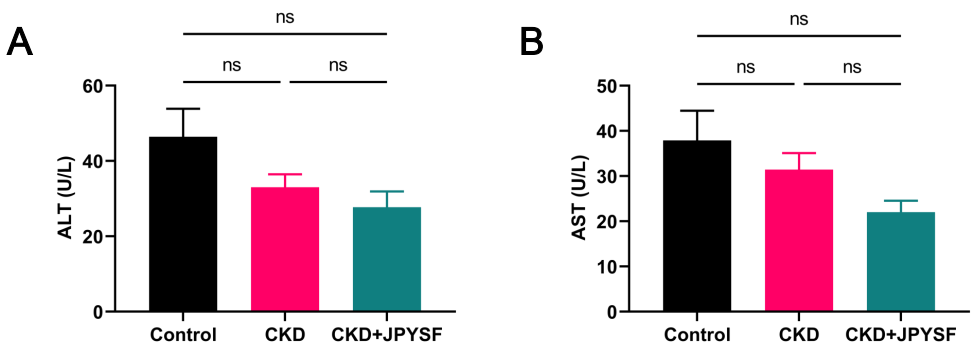


**Supplementary Figure S2.** JPYSF has no apparent effect on liver function indexes. (A) Serum alanine transaminase (ALT) levels. (B) Serum aspartate transaminase (AST) levels. Data are presented as the means ± SEM, n=5-6 rats per group. ns: no significant.


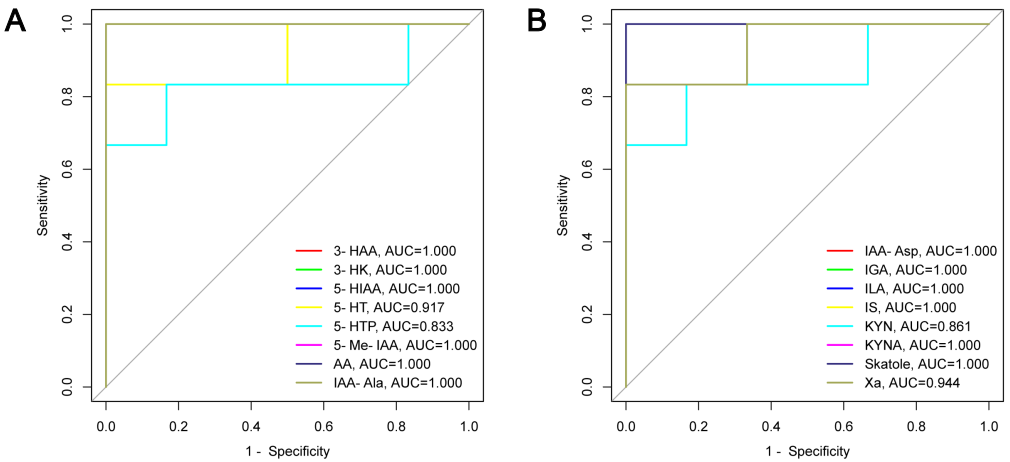


**Supplementary Figure S3.** The diagnostic values of tryptophan metabolites for CKD. (A) ROC curves of 3-HAA, 3-HK, 5-HIAA, 5-HT, 5-HTP, 5-Me-IAA, AA, and IAA-Ala for the diagnosis of CKD. (B) ROC curves of IAA-Asp, IGA, ILA, IS, KYN, KYNA, Skatole, and Xa for the diagnosis of CKD.
